# Automated Determination of Oxygen‐Dependent Enzyme Kinetics in a Tube‐in‐Tube Flow Reactor

**DOI:** 10.1002/cctc.201700811

**Published:** 2017-08-10

**Authors:** Rolf H. Ringborg, Asbjørn Toftgaard Pedersen, John M. Woodley

**Affiliations:** ^1^ Department of Chemical and Biochemical Engineering Technical University of Denmark DK-2800 Kgs. Lyngby Denmark; ^2^ EchoSkye DK-2300 Copenhagen S Denmark

**Keywords:** automated flow reactor, enzyme catalysis, kinetics, oxidation, tube-in-tube

## Abstract

Enzyme‐mediated oxidation is of particular interest to synthetic organic chemists. However, the implementation of such systems demands knowledge of enzyme kinetics. Conventionally collecting kinetic data for biocatalytic oxidations is fraught with difficulties such as low oxygen solubility in water and limited oxygen supply. Here, we present a novel method for the collection of such kinetic data using a pressurized tube‐in‐tube reactor, operated in the low‐dispersed flow regime to generate time‐series data, with minimal material consumption. Experimental development and validation of the instrument revealed not only the high degree of accuracy of the kinetic data obtained, but also the necessity of making measurements in this way to enable the accurate evaluation of high *K*_MO_ enzyme systems. For the first time, this paves the way to integrate kinetic data into the protein engineering cycle.

Selective oxidation is one of the most important transformations in synthetic organic chemistry.^1^, ^2^, ^3^ The necessity of achieving high reaction yield in such transformations makes enzymes particularly interesting as potential catalysts, on account of their exquisite selectivity in comparison with their chemo‐catalytic counterparts. However, for process application it is often difficult to reach the required reaction intensity (reaction rate and product concentration). In particular, issues such as low enzymatic activity, product/substrate inhibition, co‐factor regeneration and unfavorable thermodynamic equilibria need to be solved using biocatalytic reaction engineering. These problems are commonly investigated by studying the kinetic behavior of an enzyme under different conditions. Subsequently, using these data, the challenges in reaching the required productivity can be addressed either by protein engineering or, alternatively, process engineering to circumvent kinetic limitations. However, it would be much more effective if solutions arose from a combination of both approaches. Regardless of the approach taken, enzyme improvement naturally starts in the hands of the protein engineer who typically screens for improved enzymes using single point measurements (i.e. at a single substrate concentration) to go through many enzyme variants.^4^ In this way, protein engineering is able to deliver improved enzymes, also catalyzing the conversion of non‐natural substrates.^5^ However, single point measurements can only reveal apparent kinetic constants, such as the so‐called specificity constant (*V*_max_/*K*_M_), which can be misleading as the basis for selecting the optimal enzyme.^6^, ^7^, ^8^ At points in development at which selection is made from a smaller pool of protein variants, it would be highly desirable to comprehensively quantify the kinetics, to have an adequate basis for deciding on the best enzyme for a given reaction, and reactor configuration. Likewise, it is necessary to determine the activity of an enzyme of interest over the full range of potential operating conditions to be able to truly assess the possibilities for process implementation. On this premise, we suggest that comprehensive kinetic investigations should be integrated into the improvement cycle of an enzyme for application. In this way it would be possible to direct screening to focus on evolving improved enzymatic kinetic properties, which are ideal for process implementation. To realize such a scheme, it is necessary to develop an automated characterization system.^9^ Herein, we present one such system focused on collecting kinetic data for oxygen‐dependent enzymes.

On studying enzyme kinetics, it is important to measure initial rates at substrate concentrations well above, as well as below, the true Michaelis constant(s), to determine kinetic parameters with sufficient accuracy. In the study of oxygen‐dependent enzymes, such investigations are notoriously difficult as a result of the limited solubility of oxygen in water, and to some extent, of the concomitant limited supply rate of oxygen. The challenge of controlling the oxygen concentration leads in many cases to conducting experiments at a single oxygen concentration (usually that in water, in equilibrium with air, at 276 μm). Air saturation is however insufficient to achieve enzyme saturation for several industrially interesting oxidases^10^, ^11^, ^12^ and, in any case, it introduces uncertainty into parameter estimations. Indeed, conventional experiments can only reveal apparent Michaelis constants which are confined to the tested parameter space and should therefore be compared with great care. Likewise, oxygen supply is often carried out by bubbling air through the reaction solution. However, in doing so, it is necessary to consider the stripping of any volatile substrate(s) and product(s), as well as potential enzyme deactivation at the gas‐liquid interface.^13^ The constraint on the limited dissolved oxygen concentration in water can be alleviated by pressurizing the reactor or by using enriched air (to increase the partial pressure), whereas the interfacial effect can only be alleviated by introducing a physical barrier between the gas and the liquid.

Recently, the Teflon AF‐2400 fluoropolymer,^14^ which is characterized by high gas permeability, has been used as a membrane in the latest development of the so‐called Tube‐in‐Tube Reactor (TiTR) design^15^ which has previously proven to be useful for the supply of gaseous substrates to liquid reaction media while retaining the chemical resistance of traditional fluoropolymers.^16^, ^17^ The TiTR is made of an inner Teflon AF‐2400 tube encased within an outer PTFE tube with low oxygen permeability. A mixture of oxygen and nitrogen is supplied in the space between the two tubes, whereby the oxygen can be transferred to the liquid reaction mixture in the inner tube through the membrane. We reasoned this would make the TiTR ideal for studying the kinetics of oxygen dependent biocatalytic reactions, since the challenges of conventional systems can be avoided by creating a bubble‐free aeration system. The small dimensions of the inner tube (I.D/O.D. 230/410 μm) maximize the surface‐to‐volume ratio, which combined with the high oxygen permeability of Teflon AF‐2400, enables very high oxygen supply rates. This TiTR allows operation at dissolved concentrations of oxygen very close to the equilibrium value between the gas phase and the reaction medium, despite a low driving force (i.e. the reactor will operate at a dissolved oxygen concentration within 99 % of saturation). Additionally, by pressurizing both the inner and outer tube, the oxygen solubility in the reaction mixture can be increased proportionally. The setup therefore allows control over oxygen as a substrate in oxygen‐dependent enzyme reactions. Furthermore, the TiTR satisfies the requirement for negligible change in substrate concentration for measurement of initial rates, since oxygen can be supplied along the reactor as it is consumed. Based on this concept, a system suitable for kinetic characterization of oxygen dependent enzymes was developed by combining the TiTR with precise liquid and gas supply systems and connecting the outlet of the inner tube to a UV/Vis detector. By means of a switch valve, samples were carried from the injection loop into the detector, where the solution was subjected to flow injection analysis.

Although such a reactor is very useful for conducting oxygen‐dependent enzyme reactions (under pressure), we realized that a further development was still necessary for the meaningful collection of kinetic data. Laboratory flow reactors typically operate in the laminar flow regime with large axial dispersion, which requires steady‐state experiments. Such experiments often consume more material over a longer time period and with a lower sampling frequency than those performed in equivalent batch apparatus.^18^ Recently, a review of Taylor′s work regarding mixing and dispersion^19^ has led to the application of low dispersed flow in microreactors.^20^ This is a unique regime of laminar flow that occurs only at a microfluidic scale.^20^ In this flow regime, the radial mixing from the center of the tube to the edges is governed solely by diffusion. At the microscale, the diffusion lengths are by definition very small and this will in turn give very short radial mixing times. Low dispersed flow will therefore flatten the well‐known “tongue” profile of laminar flow, and solute concentrations will thereby only change along the length of the reactor. Consequently, the reactor can be described by plug‐flow behavior, which was used in a method recently reported by Moore and Jensen.^21^ In this method, at low residence time, steady‐state is obtained and the flow rate is subsequently ramped down. By following the conversion during the ramp, initial rate measurements (i.e. concentration‐time profiles) are possible without the need to obtain multiple steady‐states. Nevertheless, the reported Moore and Jensen method requires modification for biocatalysis. Low dispersed flow is very dependent on the diffusivity of the solutes, and the large size of enzyme catalysts translates into a two order‐of‐magnitude lower diffusivity compared to small molecules (10^−11^ cf. 10^−9^ m^2^ s^−1^).^22^, ^23^ The axial dispersion of enzymes will therefore be much more pronounced, indicating that enzymes are more dispersed along the length of the channel compared to the small molecule reactants and the resulting products. It was therefore necessary to make sure that the enzyme concentration in the entire reactor volume remained constant. This was ensured by achieving steady‐state with respect to the enzyme concentration and thereafter keeping the enzyme feed concentration constant, independent of the liquid flow rate. In this way, it was assumed that the degree of dispersion would be dependent on the diffusion coefficients of the substrate(s) and product(s) alone. The integrated combination of each of the aforementioned developments has led to the establishment of the current instrument, which now gives a novel and automated way of kinetically characterizing oxygen‐dependent enzymes, see Figure 1. The specific details of the setup are described in the Supporting Information (SI).

**Figure 1:**
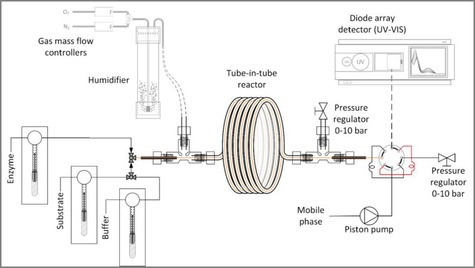
Experimental setup of the Tube‐in‐Tube Reactor. The three syringe pumps on the left deliver a liquid solution to the inner membrane tube, illustrated by the orange line. Two mass flow controllers are used to vary the gas composition in the range 5–100 % O_2_, supplied to the outer tube. The gas is wetted and heated before entering the reactor to avoid the stripping of water from the inner tube. The gas was fed through an outer tube, made of PTFE. A pressure regulator and a manometer were located at both ends of the two tubes to control the pressure, as well as to ensure an equal or higher pressure on the liquid side of the membrane.

To demonstrate the performance of the instrument, the well‐known enzyme, glucose oxidase (GOx, E.C. 1.1.3.4), was selected. The GOx enzyme catalyzes the oxidation of glucose to glucono‐δ‐lactone, using molecular oxygen (which is itself reduced to hydrogen peroxide). Following the enzymatic reaction, glucono‐δ‐lactone is spontaneously hydrolyzed to gluconic acid, which formation can be followed spectrophotometrically (see Supporting Information). The hydrogen peroxide formed is removed instantaneously by the addition of catalase, which enables its conversion into water and half the stoichiometric amount of oxygen. The removal of hydrogen peroxide forces the reaction to proceed in a unidirectional manner and also protects GOx from oxidation. GOx has been shown to follow a ping‐pong bi‐bi reaction mechanism (Scheme 1)^24^ for which a rate expression can be derived (Equation (1)).

(1) $$\frac{r}{\left[E\right]}=\frac{k_{cat}S\;O}{S\;O+K_{MO}\;S\;+K_{MS}\;O}$$

**Scheme 1:**
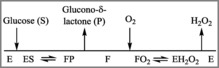
Cleveland representation of the glucose oxidase ping‐pong bi bi mechanism. E denotes the oxidized free form of the enzyme whereas F denotes the reduced form of the free enzyme.

The flow manipulation method applied to produce the equivalent batch data from the setup, requires an accurate determination of the reactor volume. Hence, initially, residence time distribution experiments were conducted to determine the volume of the reactor (155±1.8 μL, see Supporting Information). Next, the results of the flow method were compared with steady‐state operation, and it was shown that the setup indeed produces time‐series data even with the addition of a slow diffusing (bio)catalyst (see SI). Finally, to validate the enzyme kinetics measured in the TiTR, equivalent experiments to those carried out in batch by Toftgaard Pedersen and co‐workers^25^ were conducted. In the batch experiments, the setup used an aerated stirred tank reactor with adjustable oxygen/nitrogen feed. The comparison revealed an excellent correlation between the two systems and the combined results of the validation experiments confirmed that the kinetics determined using the TiTR setup are reliable (Figure 2).

**Figure 2:**
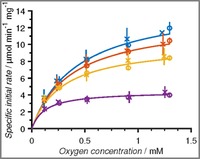
Specific initial reaction rate vs. oxygen concentration in Batch (*x*) and TiTR (o) at a glucose concentration of 400 mm (blue), 200 mm (red), 100 mm (yellow), and 25 mm (purple). Full lines represent the model fit to the TiTR results. The experiments were carried out at pH 7, 25 °C and atmospheric pressure. The batch data was scaled by a factor of 0.79 to correct for time dependent degradation of the enzyme formulation between the experiments, see Supporting Information.

The fit of Equation (1) to these data revealed a relatively high Michaelis constant of 0.52 mm for oxygen (Table 1), which is also obtained from the unsaturated enzyme kinetics observed at high glucose concentrations and atmospheric pressure (Figure 2). It is generally accepted, that to reliably quantify Michaelis constants it is necessary to measure enzyme kinetics in a sufficiently large range of substrate concentrations, comprising values that are 5‐fold (as a minimum, and preferably 10‐fold) higher and lower than the true *K*_M_. In the TiTR setup, this was achieved by increasing the operating pressure of the setup to 6 bar to increase the maximum dissolved oxygen concentration to 7.13 mm (using pure O_2_ at 25 °C). Enzyme saturation was thereby obtained even at the highest concentration of glucose (Figure 3), enabling a more reliable prediction of all the kinetic parameters (Table 1).

**Table 1:** Parameter estimations based on different experimental data. Pressure is given as absolute pressure.

Parameter	Batch reactor	TiTR	TiTR
	(1 atm)	(1 atm)	(1 atm+6 bar)
*k*_cat_ [μmol min^−1^ mg^−1^]^[a]^	17.58±0.62^[b]^	17.78±1.39	17.82±0.47
*K*_MO_ [mm]	0.45±0.04	0.51±0.09	0.52±0.03
*K*_MS_ [mm]	73.1±6.87	75.2±9.38	74.57±5.55

[a] Based on milligrams of liquid formulation [b] The batch data is scaled by a factor 0.79 to correct for time dependent degradation of the enzyme formulation between the experiments, see SI.

**Figure 3:**
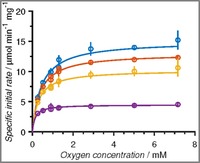
Data collected in the TiTR at 1 atm. (0.14–1.3 mm O_2_) and 6 bar (0.9–7.13 mm O_2_) at a glucose concentration of 400 mm (blue), 200 mm (red), 100 mm (yellow), and 25 mm (purple). Full lines represent the model fit. Experiments were carried out at pH 7 and 25 °C.

The TiTR setup was fully automated and computer controlled, thereby enabling characterization of an oxygen‐dependent enzyme within 24 hours with minimal manual labor. While the preparation of solutions is identical for both batch and TiTR, the batch setup requires four full days of labor. Furthermore, the small dimensions of the system make it possible to collect one initial rate measurement per 1.4 mL of reaction mixture, which is considerably less than the 150 mL required in the alternative sparged batch setup.

In summary, we have developed and validated an automated flow reactor system that rapidly and accurately determines the kinetics of oxygen‐dependent enzymes. The tool allows perfect control of the oxygen concentration in solution, which by pressurizing the system can enable values that are up to 25‐fold higher than the values achievable by using merely air under atmospheric conditions. Operation in the low dispersed flow regime allowed the generation of time‐series data with an enzymatic catalyst, despite its low diffusivity, and the resulting data were in good agreement with experiments conducted in a batch system. The system is capable of characterizing the kinetics of any enzyme within the oxidoreductase class (EC 1), for which reactions frequently result in changes to the UV‐spectra, to enable facile quantification of conversion. The application is however not limited to oxygen‐dependent enzymes alone, but can in principle be used to study many other enzymes using gaseous substrates, such as hydrogenases (using H_2_),^26^ formate dehydrogenases (using CO_2_)^27^ or methane monooxygenases (using CH_4_).^28^ The tool presented here could introduce kinetic characterization of oxidoreductases into the catalyst development cycle, where biocatalytic reaction engineering can be used to guide both process and protein engineering.^9^, ^29^ The need to improve this development cycle further is particularly important to facilitate the wider and more effective implementation of biocatalytic reactions, especially in the pharmaceutical industry.^30^

## Conflict of interest

*The authors declare no conflict of interest*.

## Supporting information

As a service to our authors and readers, this journal provides supporting information supplied by the authors. Such materials are peer reviewed and may be re‐organized for online delivery, but are not copy‐edited or typeset. Technical support issues arising from supporting information (other than missing files) should be addressed to the authors.

Supplementary
