# Supplementary material for: Automated Determination of Oxygen‐Dependent Enzyme Kinetics in a Tube‐in‐Tube Flow Reactor
Source: ChemCatChem. 2017 Aug 10;9(17):3285–8. doi: 10.1002/cctc.201700811 (PMC5768025; doi:10.1002/cctc.201700811)
Supplement: Supplementary file 1 — Supplementary [file CCTC-9-3285-s001.pdf]

Heterogeneous & Homogeneous & Bio- & Nano-
CHEMCATCHEM
CATALYSIS

Supporting Information

Automated Determination of Oxygen-Dependent Enzyme Kinetics in a Tube-in-Tube Flow Reactor

Rolf H. Ringborg+,[a, b] Asbjørn Toftgaard Pedersen+,[a] and John M. Woodley*[a]

cctc_201700811_sm_miscellaneous_information.pdf

Author Contributions

R.R. Conceptualization: Equal; Formal analysis: Lead; Methodology: Lead; Software: Lead; Writing – original draft: Equal; Writing – review & editing: Equal
A.T. Conceptualization: Equal; Data curation: Equal; Methodology: Equal; Writing – original draft: Equal; Writing – review & editing: Equal
J.W. Conceptualization: Equal; Resources: Lead; Supervision: Lead; Writing – review & editing: Equal.

MATERIALS AND METHODS

Chemicals

Glucose oxidase (EC 1.1.3.4) from *Aspergillus niger* (Novozym® 28166) was kindly supplied by Novozymes A/S (Bagsværd, DK). Catalase (EC 1.11.1.6) from bovine liver with specific activity 3172 U/mg was acquired from Sigma Aldrich (St. Louis, MO, USA). Glucose, gluconic acid, and buffer chemicals were of highest quality available from Sigma Aldrich, VWR (Radnor, PA, USA), or Thermo Fisher Scientific (Waltham, MA, USA).

Experimental setup

Reaction mixture was supplied to the setup using three identical syringe pumps (Cavro XLP 6000, TECAN®, Männedorf, Switzerland) equipped with 100 µL syringes (Figure S1, #1). The outlet of the pumps were mixed in a micro mixer (402-0005B, ASI Corp., Richmond, CA, USA) before entering the reactor. A defined gas mixture of nitrogen and oxygen was supplied using two mass-flow controllers (SmartTrak 50, Sierra Instruments, Monterey, CA, USA). The gas was prehumidified by bubbling it through a water column (constructed in-house) before the gas entered the reactor (Figure S1, #2). This ensured that the gas reached a relative humidity of 100% to avoid stripping of water from the reaction mixture in the reactor. The tube-in-tube reactor (TiTR) was constructed using an outer PTFE tube and an inner tube made of Teflon AF-2400, see below. Teflon AF-2400 is an amorphous fluoropolymer made as a copolymer of 2,2-bistriflouromethyl-4,5-diflouro-1,3-dioxole and tetrafluoroethylene.[1] Teflon AF-2400 is very permeable to oxygen, while maintaining the very high chemical resistance characteristic of PTFE. The inner tube can therefore act as a membrane allowing oxygen and nitrogen to be transferred from the gas, flowing in the space between the two tubes, to the liquid reaction mixture inside the inner tube (Figure S1, #3).

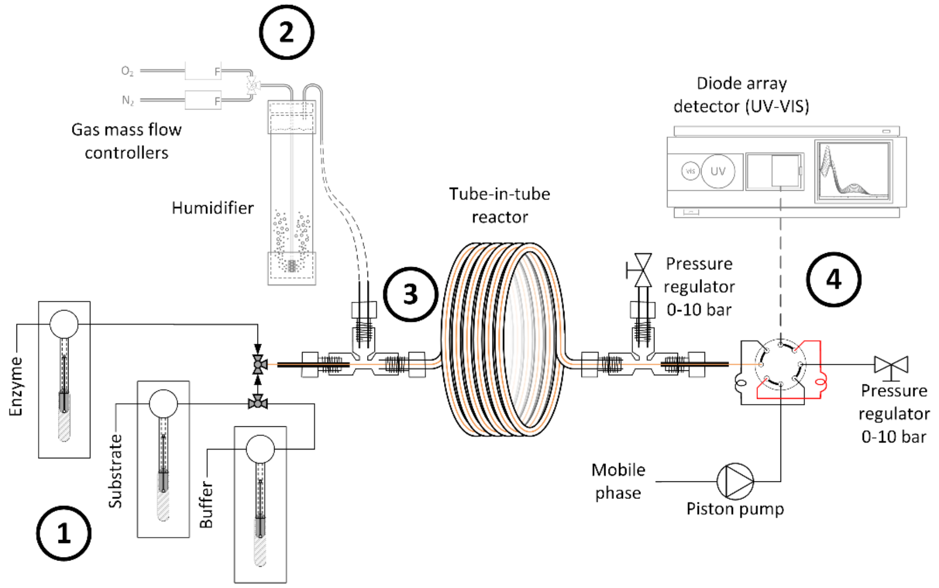

Figure S1. Tube-in-Tube reactor setup. 1) Syringe pumps for reaction mixture supply, 2) mass-flow controllers for gas mixing and supply, 3) tube-in-tube reactor, 4) UV-Vis spectrometer for analysis

The reactor was capable of being pressurized up to 10 bar on both the gas and liquid side using an adjustable gas backpressure regulator (KBP1G0D4A5A2, Swagelok, Solon, OH, USA) and an adjustable liquid

backpressure regulator (ZNF1FPK-5 VICI, Houston, TX, USA). The TiTR was submerged in a temperature-controlled water-bath. The reaction progress was followed by analyzing the composition in the liquid outlet using an UV/VIS diode array detector (G1315A, Agilent Technologies, Santa Clara, CA, USA) equipped with a 14 µL flow cell with a 1 cm path length (Figure S1, #4). Samples were injected into the flow cell using an 8-port actuated injection valve (VICI) equipped with two 5 µL injection loops. Mobile phase (deionized water buffered to pH of reaction mixture) was continuously flushed through the flow cell using an HPLC pump (Smartline 100, 10 mL ceramic pump heads, KNAUER, Berlin, Germany). The detector was not installed in-line because of dilution effects in the flow cell, i.e. the flowrate relative to the volume of the flow cell results in a system with a response time greater than the change induced by the reduction in flow rate.

Control of syringe pumps, oxygen and nitrogen flow rate, injection valve and the detector was automated using LabVIEW (National Instruments, Austin, TX, USA).

Tube-in-tube reactor

The TiTR design, with various dimensions depending on the application, has been described in detail in several scientific publications.[4–7] The TiTR used in this work was constructed from a small diameter Teflon AF-2400 tubing (I.D. 0.23 mm, O.D. 0.41 mm, Biogeneral Inc., San Diego, CA, USA), to obtain low-dispersed flow even at low residence times. The inner Teflon Af-2400 was encased by PTFE tubing (1.6 mm I.D., O.D. 3.2 mm, BOLA, Grünsfeld, Germany), practically impermeable to oxygen and nitrogen. The two tubes were each 3 meters long. The inner diameter of the outer tube was selected to provide a relatively large cross sectional area. This ensured that the gas-side pressure drop across the reactor was minimal and enabled the precise control of the gas-side pressure to ensure a uniform dissolved oxygen concentration along the entire length of the reactor. On the other hand, a liquid-side pressure drop of up to 1 bar could not be avoided. However, as long as a liquid-side pressure is maintained well above the gas-side pressure was maintained no gas bubbles are formed in the liquid stream, and the dissolved oxygen concentration could be controlled purely by the gas-side partial pressure of oxygen.

The construction of the TiTR is straightforward and possible with of-the-shelf components. The most critical step was to ensure an air and liquid tight inlet and outlet connection. The T-connection merging the gas and liquid lines into the tube-in-tube reactor were constructed with the following fittings (IDEX-HS, Lake Forrest, IL, USA): T-connector for 1/8” tubing (P-713), which included nuts for 1/8” tubing (P-335) and ferrules for 1/8” tubing (P-300). Furthermore: nuts for 1/16” tubing (XP-201), ferrules for 1/16” tubing (P-200), and tube sleeve (I.D. 0.46 mm, O.D. 1.6 mm, F-243). The final assembly can be seen in Figure S2.

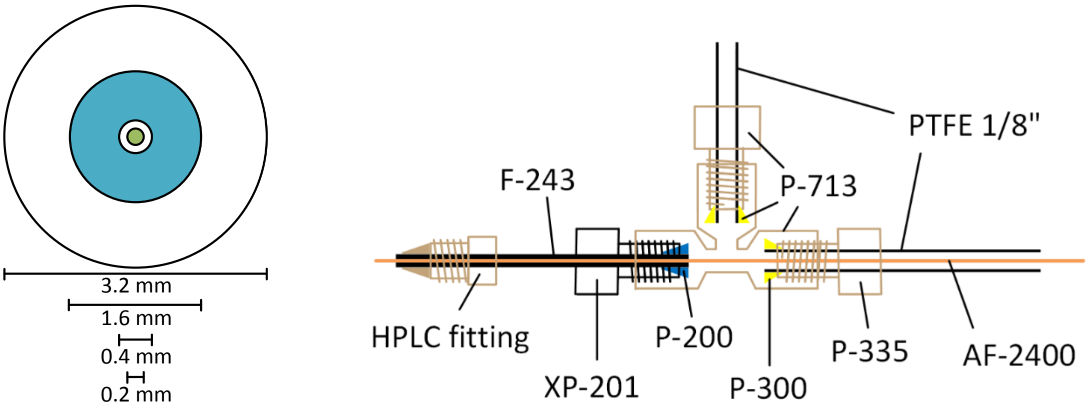

Figure S2. Left: Cross-section of the tube-in-tube reactor, where green indicates liquid media and blue gas phase. Right: Final assembly of either end of the TiTR reactor.

Protocol

Pumps were initially primed with the designated substrate, buffer and enzyme solutions, and were thereafter set to run continuously to reach steady state. In parallel, the mass-flow controllers were set to produce a defined mixture of oxygen and nitrogen. To ensure steady state, the system was set to wait a total of 3 residence times of 2 min for all set points. After which a sample was injected into the detector, and the ramp method initiated. The method gradually lowers the flow rate, without changing the inlet concentration any component, from the initial steady state value to increase the residence time. By analyzing the output of the reactor, precise time series data can be obtained, as described by Moore and Jensen.[8] They defined a linear relationship between instantaneous residence time and real time, with a parameter α as the slope. α was set to 0.5 in all experiments reported in this work and the ramp in flow rate was run for 15 min. (real time) generating data from a residence time of 2 min. to 10 min. Samples were collected every minute resulting in 11 samples for each initial rate experiment. The flow rate for each syringe pump and percentage of oxygen in the gas was controlled by LabVIEW, which automatically changed the set-point according to a predefined list of set-points.

Samples were analyzed on the diode array UV/Vis detector. Before a sample was injected, the detector was balanced and zeroed. Once it reported ready, the injection valve was turned to inject a sample using the flow of the mobile phase. Thereafter, spectral data was collected over time with a frequency of 20 Hz whereby 3-dimensional (time-wavelength-absorbance) data was obtained. The absorbance was measured from 210 to 600 nm with a slit width of 4 nm and a step width of 1 nm.

To characterize an enzyme, three stock solutions were made – one containing 0.5 mg/mL glucose oxidase in buffer, one containing buffer, and one containing 500 mM glucose in buffer. All solutions were buffered to pH 7.0 using 100 mM potassium phosphate buffer. The enzyme solution was pumped at a constant ratio of 1/5 of the total flow rate through the reactor, while the ratios of buffer and glucose solutions were varied according to the glucose concentration set-point. The total gas flow was kept constant at 1 NL/min, while the volume fraction of oxygen in the gas was varied from 5-100%. For the experiments shown in this paper the reactor was left to run at atmospheric pressure and in a separate experiment pressurized to 6 bars.

Data handling and parameter estimation was performed in MATLAB (MathWorks, Natick, MA, USA). Initially, the raw data from LabVIEW was converted to concentrations by using a calibration model. A linear model was fitted to the concentration-time data to obtain the initial rate as the slope. The data was discarded if the linear model was not describing the data sufficiently well ($R^2 < 0.9$). The parameters of the enzyme kinetic model (ping pong bi bi) was estimated using non-linear least square regression taken into account the uncertainty of the initial rate data.

ANALYSIS OF REACTION COMPONENTS

The experimental setup requires that one or more reaction species contains a UV chromophore in order to follow the reaction using the UV/Vis detector. The oxidation of glucose to glucono-δ-lactone using glucose oxidase was chosen as the demonstration system. Glucose does not absorb UV light to any great extent and can therefore not be quantified reliably. On the other hand, gluconic acid, which is formed upon instantaneous hydrolysis of glucono-δ-lactone, has a much higher molar absorption coefficient, making it ideal for detection using the chosen detection method (Figure S3).

The obvious difference in absorbance strength between glucose and gluconic acid makes quantification of the gluconic acid concentration straightforward using a single wavelength. 210 nm was chosen as the preferred wavelength. The absorbance-time data was integrated to obtain a peak area, which was correlated with a gluconic acid concentration as seen in Figure S3. Glucose (and the enzyme) also absorb

light at 210 nm, however, the absorbance is small and the concentration of glucose is almost constant. The contribution of such can therefore be neglected, since we are solely interested in the rate of reaction and not the absolute gluconic acid concentration.

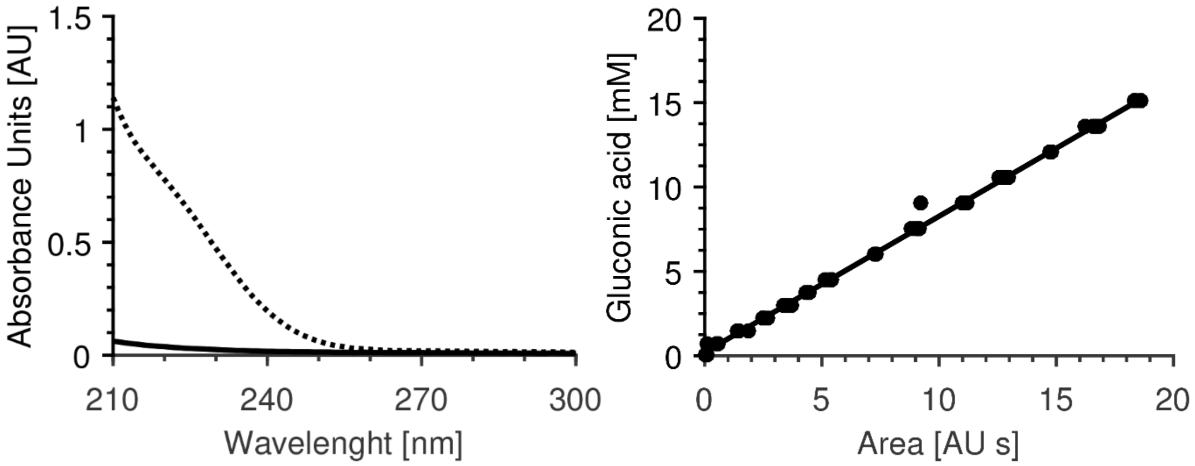

Figure S3. Left: Absorbance spectra of 10 mM glucose (solid line) and 10 mM gluconic acid (dotted line). Right: Calibration of gluconic acid at a wavelength λ = 210 nm

REACTOR VOLUME

It is very important to know the precise volume of the reactor in order to determine the correct residence time and therefore reaction rate. It is possible to calculate the volume based on the inner diameter of the inner tube and the dead volume of the fittings. However, this is rather unprecise because the tolerance of the inner tubing diameter is typically ±25 µm or more, which leads to a variation of volume for our case of ± 28.5 µL. The liquid volume was therefore determined based on residence time distribution experiments (Figure S4). The volume was determined to be 155 ± 1.8 µL by the method described in the textbook of Fogler[9].

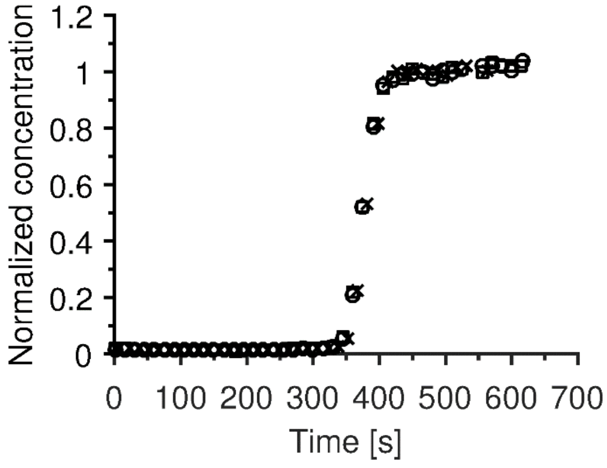

Figure S4. Residence time distribution experiments. Three repetitions of a step change response at a flow rate of 25 µL/min.

COMPARISON OF STEADY-STATE EXPERIMENTS WITH FLOW RAMP MEASUREMENTS

The validity of the ramp method for generating time-series data has been thoroughly validated by Moore and Jensen[8]. However, to verify that the method was reliable also for enzyme catalyzed reactions, data that has been generated using the ramp method was therefore compared with steady state experiments (Figure S5). The steady-state data was collected after 4 residence times. The results show that there is little to no difference between the two sampling methods, indicating that that the low dispersed flow condition was met.

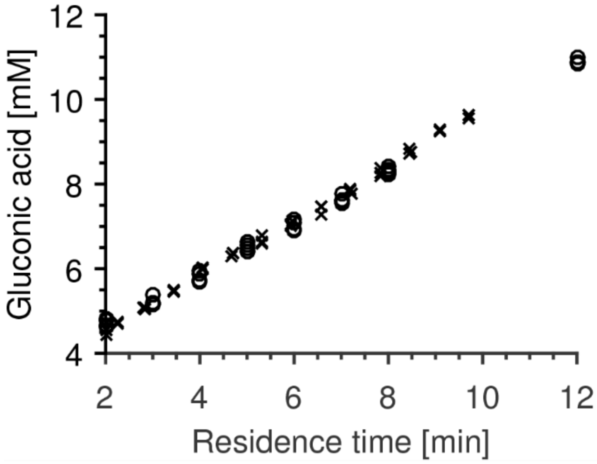

Figure S5. Comparison of running the setup in a steady state (o) mode and ramp mode (x), experiments were carried out with 0.1 mg/mL GOx enzyme, 100 mM glucose, 0.52 mM O_2 and with 100 mM Potassium phosphate buffer at pH 7.

OXYGEN TRANSFER RATE

Teflon AF-2400 is extremely oxygen permeable with a permeability of 990-1600 Barrer[2,3] (1 Barrer = cm^3 cm (s^{-1} cm^{-2} (cm Hg))$^{-1}$) compared to an oxygen permeability of traditional PTFE of 4.2 Barrer[2]. Combined with the very large surface area to volume ratio for the reactor (17000 m^2/m^3) this results in very high oxygen transfer rates (corresponding to a volumetric mass transfer coefficient (k_La) of 16000-26000 h^{-1}). The high mass transfer coefficient means that the TiTR can be operated so that the dissolved oxygen concentration is within 99% of the oxygen solubility at the given temperature and partial pressure of oxygen, even with reaction rates of more than 5 mmol/L/min. Reaction limited operation of the setup was confirmed by varying the enzyme concentration from 50 mg/L to 390 mg/L as seen in Figure S6. From the figure, it is clear that there is a linear dependency between enzyme concentration and rate of reaction, as one would expect if mass-transfer does not influence the rate of reaction.

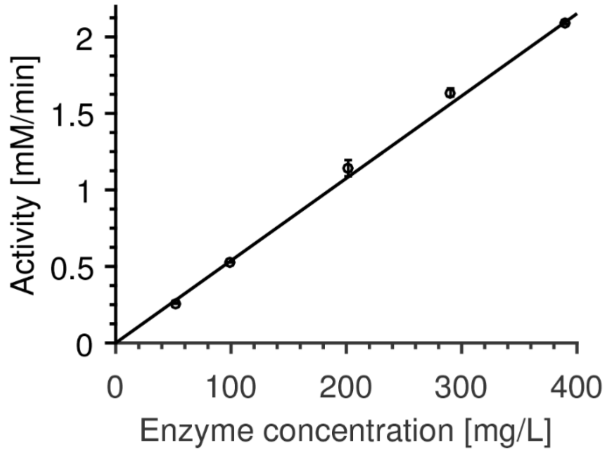

Figure S6. Activity as a function of enzyme concentration at an oxygen concentration of 0.52 mM and a glucose concentration of 100 mM.

RESCALING OF BATCH DATA

The kinetic data recorded using the TiTR was compared to previously published batch data[10]. The enzyme formulation used was identical to the formulation used in the previous study, however, due to the time between experiments, approximately 10 months, the activity of the formulation decreased. The batch data previously published was therefore scaled with a constant factor of 0.79 to account for the time dependent deactivation. Table S1 shows the activity of glucose oxidase at the point in time for the experiments.

Table S1. Activity of enzyme formulation when batch kinetic data was collected compared to when TiTR data was collected.

	**Specific activity[a] (µmol/min/mg[b])**	**Relative Activity**
**Batch experiments**	14.4±0.1	100%
**TiTR experiments**	11.4±0.2	79.4%

[a] 400 mM glucose, 12.5 mg/L GOx, 5 mg/L catalase, pH 7.0, stirred reactor, bubbling with pure oxygen.
[b] Based on weight of liquid enzyme formulation.

References

[1] P. R. Resnick, W. H. Buck, *Mod. Fluoropolymers* **1997**, 397–419.

[2] S. M. Nemser, I. C. Roman, *Perfluorodioxole Membranes*, **1991**, US5051114.

[3] I. Pinnau, L. G. Toy, *J. Memb. Sci.* **1996**, *109*, 125–133.

[4] M. O'Brien, I. R. Baxendale, S. V. Ley, *Org. Lett.* **2010**, *12*, 1596–1598.

[5] A. Polyzos, M. O'Brien, T. P. Petersen, I. R. Baxendale, S. V. Ley, *Angew. Chemie - Int. Ed.* **2011**, *50*, 1190–1193.

[6] B. Tomaszewski, A. Schmid, K. Buehler, *Org. Process Res. Dev.* **2014**, *18*, 1516–1526.

[7] M. O'Brien, N. Taylor, A. Polyzos, I. R. Baxendale, S. V. Ley, *Chem. Sci.* **2011**, *2*, 1250–1257.

[8] J. S. Moore, K. F. Jensen, *Angew. Chem. Int. Ed. Engl.* **2014**, *53*, 470–473.

[9] H. S. Fogler, *Elements of Chemical Reaction Engineering*, Pearson Education, Inc, **2005**.

[10] A. Toftgaard Pedersen, T. Carvalho, E. Sutherland, G. Rehn, R. Ashe, J. M. Woodley, *Biotechnol. Bioeng.* **2017**.
